# Efficacy of Blinatumomab in Pediatric Acute Lymphoblastic Leukemia: A Systematic Review and Meta-Analysis of Randomized Controlled Trials

**DOI:** 10.7759/cureus.86260

**Published:** 2025-06-18

**Authors:** Oboseh J Ogedegbe, Olanipekun L Ntukidem, Gautham Varun Krishna Mohan, Shahnawaz Shah, Abdallah A Riyalat, Calvin R Wei, Syed Ahmar Hussain, Danish Allahwala

**Affiliations:** 1 Internal Medicine, Lifeway Medical Center, Abuja, NGA; 2 Internal Medicine, Trinity Health, Ann Arbor, USA; 3 Internal Medicine, Tirunelveli Medical College, Tirunelveli, IND; 4 Acute Medicine, University Hospital of Bristol and Weston National Health Service (NHS) Trust, Weston-super-Mare, GBR; 5 Pediatric Medicine, Sidra Medicine, Doha, QAT; 6 Research and Development, Shing Huei Group, Taipei, TWN; 7 Padiatrics, Ashfaq Memorial Hospital, Karachi, PAK; 8 Nephrology, Fatima Memorial Hospital, Karachi, PAK

**Keywords:** acute lymphoblastic leukemia, blinatumomab, immunotherapy, meta-analysis, pediatric oncology

## Abstract

Blinatumomab, a bispecific T-cell engager antibody, has emerged as a promising immunotherapeutic agent for pediatric acute lymphoblastic leukemia (ALL), yet comprehensive evidence regarding its efficacy remains limited. This systematic review and meta-analysis aimed to evaluate the therapeutic outcomes of blinatumomab in children with ALL. A comprehensive literature search was conducted across PubMed, EMBASE, Web of Science, and Cochrane Library databases from inception to May 2025, using terms related to blinatumomab, ALL, and pediatric populations. Studies comparing blinatumomab with chemotherapy or placebo in children and adolescents with B-cell ALL were included. Three randomized controlled trials met the inclusion criteria and were analyzed using random-effects models. Quality assessment was performed using the Cochrane Risk of Bias Tool (RoB 2, Cochrane Collaboration, London, UK). The meta-analysis demonstrated significantly superior outcomes with blinatumomab compared to chemotherapy alone. Overall survival was significantly higher in the blinatumomab group, with an odds ratio of 1.90 (95% CI: 1.28-2.82). Event-free survival showed even greater improvement with an odds ratio of 2.97 (95% CI: 2.13-4.13). Additionally, the cumulative incidence of relapse was substantially lower in patients receiving blinatumomab, with an odds ratio of 0.26 (95% CI: 0.18-0.39). No evidence of heterogeneity was observed across studies for any outcome measure. These findings suggest that blinatumomab offers significant therapeutic advantages over conventional chemotherapy in pediatric patients with ALL, providing improved survival outcomes and reduced relapse rates. The results support the integration of blinatumomab into treatment protocols for children with ALL, particularly those at high risk of relapse or with refractory disease.

## Introduction and background

Acute lymphoblastic leukemia (ALL) is the most common pediatric cancer, accounting for approximately 25% of all childhood malignancies and representing a major cause of cancer-related morbidity and mortality in children worldwide [1]. Over the past decades, advances in risk stratification, supportive care, and multi-agent chemotherapy have significantly improved the prognosis of pediatric ALL, with long-term survival rates exceeding 85% in high-income countries [2]. However, relapse remains a critical challenge, especially in children with high-risk disease, minimal residual disease (MRD) positivity, or refractory ALL, for whom conventional therapies are often insufficient to achieve sustained remission [3].

Blinatumomab, a bispecific T-cell engager antibody construct, has emerged as a promising immunotherapeutic agent in the treatment landscape for relapsed or refractory B-cell precursor ALL (R/R B-ALL) [4]. It functions by simultaneously binding CD19 on leukemic B-cells and CD3 on T-cells, thereby redirecting T-cell cytotoxicity toward malignant B-cells [5]. The unique mechanism of action of blinatumomab offers a targeted approach to eliminating leukemic cells with minimal off-target toxicity. In adult patients with R/R B-ALL, blinatumomab has demonstrated superior efficacy compared to conventional chemotherapy, leading to its regulatory approval [4-5]. Its use has also been extended to pediatric patients, particularly those with MRD-positive disease or R/R B-ALL, where it has shown encouraging results in inducing remission and improving survival outcomes [6].

Yu et al. performed a meta-analysis to assess the efficacy of blinatumomab in treating adults with R/R B-ALL [7]. Marrapodi et al. conducted a systematic review and meta-analysis of four studies to better clarify the role of blinatumomab in the treatment of pediatric ALL, particularly in the context of ongoing efforts to minimize treatment-related toxicity and enhance long-term survival outcomes [8].

A comprehensive synthesis of the available evidence is essential to better understand the role of blinatumomab in the pediatric ALL treatment paradigm, particularly as efforts continue to reduce treatment-related toxicity and improve long-term survival. This systematic review and meta-analysis aim to evaluate the efficacy and safety of blinatumomab in children with ALL by pooling data from existing clinical studies.

## Review

Methodology

This meta-analysis was performed as per the guidelines of the Preferred Reporting Items for Systematic Reviews and Meta-Analyses (PRISMA).

Literature Search

Two authors independently conducted searches in online databases, including PubMed, EMBASE, Web of Science, and the Cochrane Library, from the inception of the databases to May 5, 2025. The search strategy utilized a combination of Medical Subject Headings (MeSH) terms and free-text keywords related to "blinatumomab", "acute lymphoblastic leukemia", "ALL", "relapsed", "refractory", and "pediatric" or "children". Boolean operators such as "AND" and "OR" were applied to refine the results. Additionally, the reference lists of the included studies and relevant reviews were manually screened to identify any additional eligible articles not captured through the database search. We included studies irrespective of the language.

Eligibility Criteria

We included studies that involved children or adolescents with B-ALL and compared blinatumomab with a placebo or other treatment group. We reported at least one of the outcomes assessed in this meta-analysis. We excluded case reports, case series, and review papers. We also excluded animal and in vitro studies. Additionally, we excluded studies that lack a comparison group. After the removal of duplicate records, all titles and abstracts were independently screened by two reviewers to assess eligibility based on the predefined inclusion and exclusion criteria. Full-text articles were retrieved for studies that met the inclusion criteria or where eligibility was unclear during initial screening. Discrepancies between reviewers were resolved through discussion or consultation with a third reviewer. The final selection included studies that provided sufficient clinical outcome data for quantitative synthesis.

Data Extraction

An information extraction spreadsheet was created specifically for this study, capturing details such as the study title, lead author, publication year, study design, country of origin, patient demographics (including age, sex, and sample size), dosage administered in the intervention group, duration of follow-up, and reported outcome measures.

Quality Assessment

Two reviewers independently evaluated the methodological quality of all included randomized controlled trials (RCTs) with the Cochrane Risk of Bias Tool (RoB 2, Cochrane Collaboration, London, UK). This tool assesses the risk of bias in five domains: bias introduced by the randomization process, deviations from intended interventions, missing outcome data, outcome measurement, and selection of reported outcomes. Each domain was classified as having a low, some concerns, or high risk of bias. Disagreements among reviewers were resolved through discussion or by engaging a third reviewer. The overall risk of bias for each study was summarized and represented graphically.

Data Analysis

All statistical analyses were performed using RevMan 5.4 (Review Manager, Cochrane Collaboration, London, UK). For binary outcomes, odds ratios (OR) with 95% confidence intervals (CI) were calculated. A p-value less than 0.05 was considered significant. We used a random-effects model when calculating the pooled estimate to account for the variation among the studies. Statistical heterogeneity will be assessed using the chi-square test (p<0.10 indicating significant heterogeneity) and quantified using the I² statistic, where 0-25% indicates low heterogeneity, 26-50% moderate, 51-75% substantial, and >75% considerable heterogeneity.

Results

**Figure 1:**
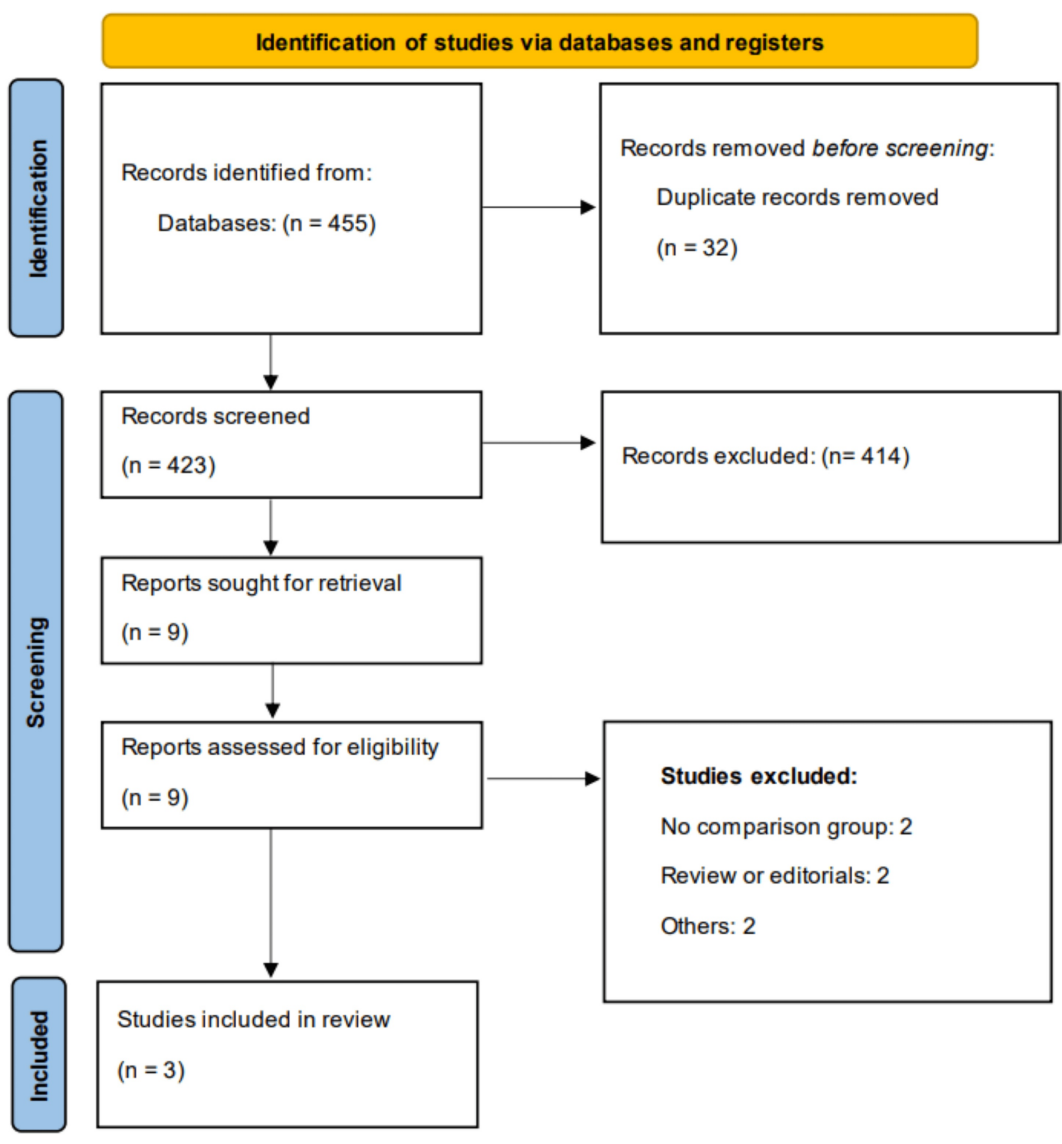
PRISMA flowchart (study selection process) PRISMA: Preferred Reporting Items for Systematic reviews and Meta-Analyses

**Figure 2:**
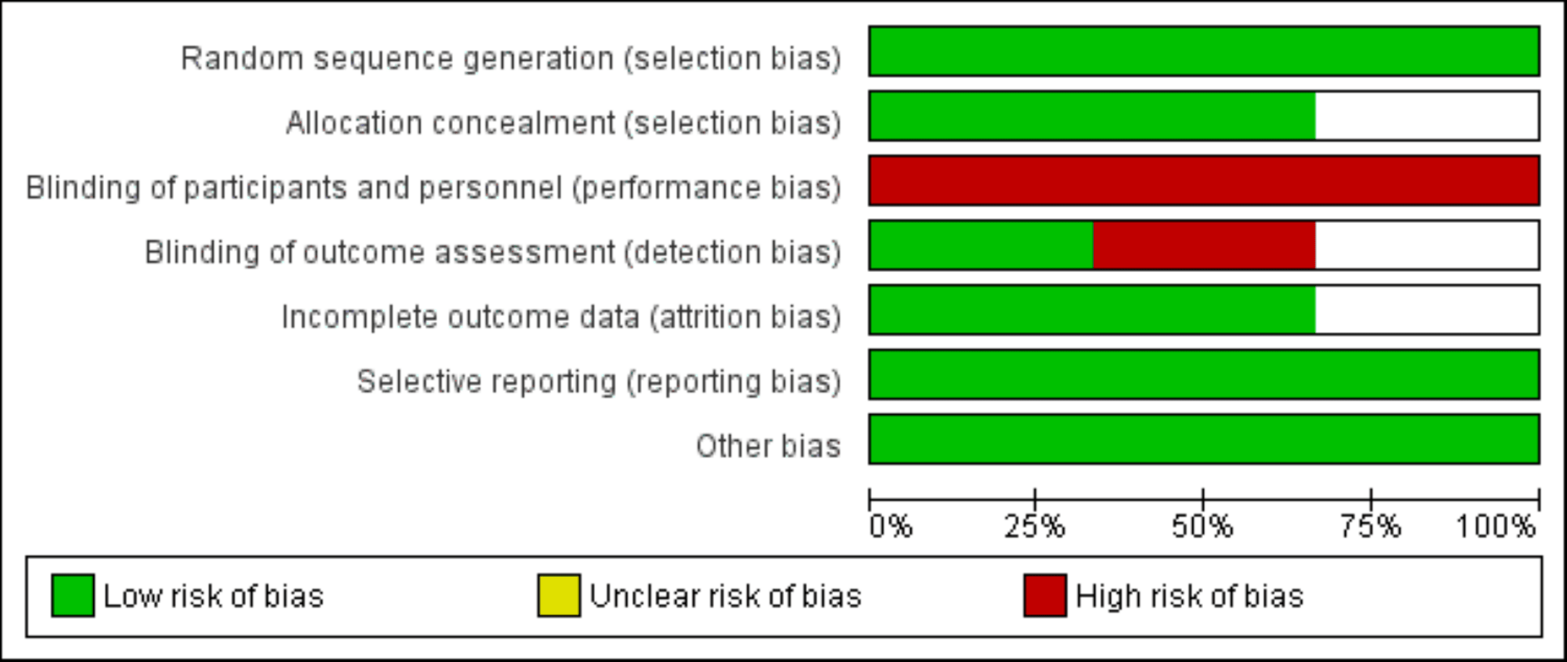
Quality assessment of included studies

We found 455 studies while scanning web databases. Following an initial examination, we identified nine studies that were eligible for detailed screening. Finally, three studies were considered in this meta-analysis. Figure 1 depicts the PRISMA flowchart for study selection. Table 1 summarizes the characteristics of the included studies. Two of the included studies were conducted in Italy and the United States, while one study was a multinational trial conducted in different countries. All studies used the same dose of blinatumomab. Figure 2 presents the quality assessment of the included studies.

**Table 1:** Included study characteristics

Author	Year	Region	Follow-up duration	Groups	Sample size	Dose of blinatumomab	Mean age (years)	Female
Brown et al. [9]	2019	Multicenter	34.8 months	Blinatumomab plus chemo	105	15 ug/m2/day	6	48
				Chemo	103		6	49
Gupta et al. [10]	2024	United States	30 months	Blinatumomab plus chemo	718	15 ug/m2/day	4.4	350
				Chemo	722		4.2	332
Locatelli et al. [11]	2021	Italy	22.4 months	Blinatumomab plus chemo	54	15 ug/m2/day	6	24
				Chemo	54		5	32

Meta-Analysis of Outcomes

**Figure 3:**
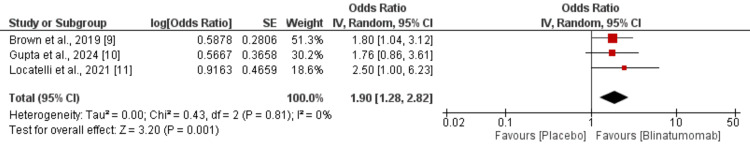
Comparison of overall survival between two groups SE: standard error, CI: confidence interval, IV: inverse variance, df: degrees of freedom [9-11]

Overall survival: Pooled data from the three RCTs demonstrated a significantly higher overall survival in the blinatumomab group compared to the chemotherapy group, with an odds ratio of 1.90 (95% CI: 1.28-2.82) as shown in Figure 3. The analysis revealed no evidence of heterogeneity among the included studies, indicating that blinatumomab provides a survival advantage over chemotherapy in pediatric patients with ALL.

**Figure 4:**
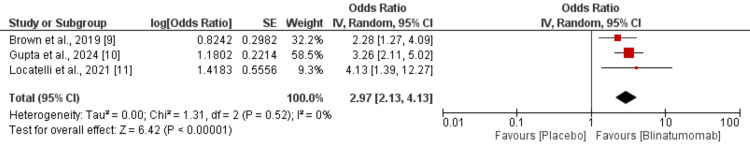
Comparison of event-free survival between two groups SE: standard error, CI: confidence interval, IV: inverse variance, df: degrees of freedom [9-11]

Event-free survival: Based on combined data from the three RCTs, a significantly higher event-free survival was reported in the blinatumomab group compared to the chemotherapy group, with an odds ratio of 2.97 (95% CI: 2.13-4.13) as shown in Figure 4. The analysis showed no evidence of heterogeneity among the included studies.

**Figure 5:**
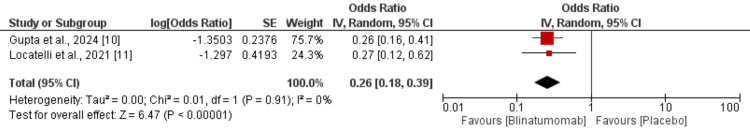
Comparison of CIR between two groups CIR: cumulative incidence of relapse, SE: standard error, CI: confidence interval, IV: inverse variance, df: degrees of freedom [10-11]

Cumulative incidence of relapse (CIR): Based on combined data from the two RCTs, a significantly lower CIR was reported in the blinatumomab group compared to the chemotherapy group, with an odds ratio of 0.26 (95% CI: 0.18-0.39) as shown in Figure 5. The analysis showed no evidence of heterogeneity among the included studies.

Discussion

Our meta-analysis evaluated the efficacy and safety of blinatumomab in children with ALL using data from three RCTs. We assessed the therapeutic effects of blinatumomab in relation to overall survival, event-free survival, and CIR. A pooled analysis of studies revealed that overall survival and event-free survival were significantly improved in children who received blinatumomab. Additionally, CIR was lower in patients receiving blinatumomab compared to patients receiving chemotherapy alone. This demonstrates that blinatumomab offers therapeutic benefits over chemotherapy in pediatric patients with ALL. The meta-analysis conducted by Chen et al., which included two RCTs and ten single-arm studies, demonstrated that blinatumomab exhibits strong therapeutic effectiveness and a favorable safety profile with minimal adverse events in pediatric patients with relapsed or R/R B-ALL [12].

The initial use of blinatumomab in treating pediatric patients was reported in a small cohort of children with relapsed ALL following allogeneic hematopoietic stem cell transplantation (HSCT). In this study, Handgretinger et al. demonstrated that blinatumomab-induced donor T-cell activation led to complete remission (CR) in three pediatric patients with post-transplant relapsed ALL [13]. Until two years ago, only one Phase I/II clinical trial had been published assessing the use of blinatumomab in pediatric patients with R/R B-ALL. This study included children with ≥25% bone marrow blasts and showed that blinatumomab, used as a single agent, possessed strong anti-leukemic activity. Among 70 treated patients, 27 achieved CR within the first two cycles, and 14 reached complete MRD remission [14].

Following promising results from earlier clinical trials demonstrating the effectiveness of blinatumomab in adults with refractory or MRD-positive B-ALL [15-16], multiple recent randomized clinical trials have explored the benefits of integrating blinatumomab into chemotherapy protocols. In a study by Litzow et al., the addition of four cycles of blinatumomab to four cycles of consolidation chemotherapy significantly enhanced relapse-free survival in adults with MRD-negative B-ALL. The three-year relapse-free survival rate was 80% for the group receiving both blinatumomab and chemotherapy, compared to 64% for those receiving chemotherapy alone (hazard ratio for relapse or death: 0.53; 95% CI: 0.32-0.87) [17]. Similarly, findings from the COG AALL1331 trial revealed that adding blinatumomab improved treatment outcomes in pediatric patients, regardless of whether they were at high or low risk of relapse [18].

The goal of blinatumomab therapy is to establish favorable conditions for HSCT, which is essential for achieving sustained remission. Compared to standard chemotherapy, a greater number of patients receiving blinatumomab proceeded to transplant, likely due to its ability to induce higher MRD negativity and fewer adverse events. The reduced likelihood of relapse in the blinatumomab group aligns with previous evidence indicating that achieving MRD remission prior to allogeneic HSCT enhances post-transplant outcomes in pediatric patients with ALL [19,20].

Patients with an average risk of relapse who received blinatumomab and chemotherapy were significantly more likely to experience sepsis and catheter-related infections of grade 3 or higher during overall protocol therapy compared to those receiving chemotherapy alone (14.8% vs. 5.1%; p<0.001) [10]. Notably, this increased infection risk was not attributable to events occurring during blinatumomab cycles but rather to events occurring during subsequent treatment phases, suggesting that B-cell aplasia, a known consequence of CD19-directed therapy, may compromise immune function beyond the immediate treatment period. While infections of grade 4 or higher remained rare, the duration of B-cell aplasia and its long-term effects on infection risk remain poorly characterized [10]. The reduced likelihood of relapse in the blinatumomab group aligns with previous evidence indicating that achieving MRD remission prior to allogeneic HSCT enhances post-transplant outcomes in pediatric patients with ALL. Consequently, while blinatumomab as consolidation treatment offers a promising alternative to traditional chemotherapy before transplantation, careful consideration of enhanced supportive care measures, including potential intravenous immune globulin or antimicrobial prophylaxis, may be warranted to mitigate the increased infectious complications in this vulnerable population.

The present meta-analysis has several important limitations that should be acknowledged. Firstly, only three RCTs met the inclusion criteria, which limits statistical power and generalizability of conclusions. This small number limits our ability to detect potential sources of heterogeneity and may not capture the full spectrum of clinical scenarios in which blinatumomab is utilized. Secondly, the insufficient number of studies and lack of disaggregated data prevented meaningful subgroup analyses by age categories, disease characteristics, risk stratification, or geographic regions, which would have provided valuable insights into treatment effectiveness across different patient populations. Thirdly, one included trial enrolled both pediatric patients and young adults, and the published data did not allow for the extraction of pediatric-specific outcomes, potentially introducing age-related confounding factors. Additionally, the small study number precluded adequate assessment of publication bias, raising concerns about potential unpublished negative results.

## Conclusions

This systematic review and meta-analysis provide compelling evidence supporting the therapeutic superiority of blinatumomab over conventional chemotherapy in pediatric ALL. The pooled analysis of three RCTs demonstrated significantly improved overall survival, event-free survival, and reduced CIR in children receiving blinatumomab treatment. These findings suggest that blinatumomab represents a valuable addition to the pediatric ALL treatment armamentarium, particularly for patients with high-risk disease, MRD positivity, or relapsed/refractory presentations. The consistently favorable outcomes across all evaluated endpoints, combined with the absence of statistical heterogeneity between studies, strengthen confidence in these results. However, the limited number of available studies highlights the need for additional large-scale RCTs to further validate these findings and explore optimal treatment protocols. Future research should focus on identifying specific patient subgroups most likely to benefit from blinatumomab therapy and determining the most effective integration strategies within existing treatment paradigms to maximize therapeutic benefits while minimizing treatment-related toxicity.
